# Economic insecurity, decision fatigue, and positive wellbeing in digitally mediated financial decision environments

**DOI:** 10.3389/fpsyg.2026.1858489

**Published:** 2026-08-19

**Authors:** Xueyan Yu

**Affiliations:** School of Accounting, Shanghai Lixin University of Accounting and Finance, Shanghai, China

**Keywords:** decision fatigue, digital finance, economic insecurity, financial decision making, intertemporal choice, positive wellbeing, self-regulation, subjective wellbeing

## Abstract

**Introduction:**

Economic insecurity is not only an objective condition of limited resources but also a subjective state of anticipated vulnerability that may shape how people evaluate and regulate consequential financial choices. This study examines whether decision fatigue helps explain the association between economic insecurity and lower subjective and positive wellbeing in digitally mediated financial decision environments.

**Methods:**

The empirical design contained two complementary components. European Social Survey (ESS) Round 6 data (*N* = 43,782 respondents nested in 29 countries) were used to test the general cross-national association between economic insecurity and subjective wellbeing. A three-wave survey of adult digital finance users in China (*N* = 1,512) tested the proposed mediation mechanism, with economic insecurity measured at T1, financial decision fatigue at T2, and WHO-5 positive wellbeing at T3.

**Results:**

In the ESS, economic insecurity was consistently associated with lower happiness and life satisfaction after demographic, socioeconomic, health-related, and country-level differences were taken into account. In the three-wave survey, T1 economic insecurity prospectively predicted greater T2 decision fatigue, and T2 decision fatigue predicted lower T3 WHO-5 positive wellbeing. The bootstrapped indirect effect was significant and remained stable across alternative outcome measures, alternative insecurity operationalizations, ordered-response models, and common-method diagnostics. The indirect pathway was stronger among frequent digital finance tool users and participants with heavier debt burdens.

**Discussion:**

The findings support a cautious longitudinal interpretation: economic insecurity is associated with lower positive wellbeing partly because it is linked to a more fatiguing pattern of financial decision engagement. The results clarify decision fatigue as a plausible psychological mechanism while avoiding a definitive causal claim and do not imply that digital finance is inherently harmful.

## 1 Introduction

Contemporary financial life increasingly unfolds through digitally mediated financial decision environments. In this article, this term refers to everyday financial contexts in which individuals use mobile banking, digital credit, budgeting applications, investment platforms, robo-advisory interfaces, or recommendation-supported financial tools to monitor, compare, and make financial choices. The term does not imply that every decision is fully algorithmically determined, nor does it treat digital tool use as equivalent to algorithmic decision intensity. Rather, it identifies a broad decision environment in which financially consequential information and choices are made more frequent, more visible, and more continuously accessible (Belanche et al., 2019; Carlin et al., 2023; Zhao et al., 2024; Lei et al., 2023). This distinction is important because the psychological focus of the present study is not technology itself, but the burden of repeated financial judgment under perceived economic vulnerability.

Economic insecurity has been consistently linked to poorer mental health, reduced life satisfaction, and lower wellbeing (Rohde et al., 2016; Kopasker et al., 2018; Ryu and Fan, 2023; Basyouni and El Keshky, 2021). This literature shows that financial hardship matters not only because of objective deprivation, but also because of the subjective appraisal of instability, unpredictability, and limited coping capacity (Adler et al., 2000; Singh-Manoux et al., 2003; Smith et al., 2012). A person who perceives their financial life as fragile may experience even ordinary economic choices as consequential, effortful, and difficult to reverse. Such subjective insecurity can maintain vigilance toward future expenses, debt obligations, income instability, and potential losses (Haushofer and Fehr, 2014; Mani et al., 2013). The resulting burden may be especially important in financial environments that invite frequent monitoring and comparison.

The outcome of interest in this study is positive wellbeing. The public-data component uses happiness and life satisfaction, and the three-wave survey uses the WHO-5 wellbeing index. These indicators capture subjective and hedonic aspects of wellbeing, including life evaluation, positive affective functioning, vitality, and energy. They do not fully represent the broader eudaimonic construct of psychological wellbeing proposed in traditions such as Ryff's model, which also includes autonomy, personal growth, environmental mastery, purpose in life, and positive relations (Ryff, 1989; Ryff and Keyes, 1995). For this reason, the manuscript uses the term positive wellbeing when referring to the empirical outcome. The broader term psychological wellbeing is used only when discussing the wider literature on positive psychological functioning (Diener et al., 1999; Keyes, 2002; Topp et al., 2015; Huppert and So, 2013).

Despite substantial evidence that economic insecurity is associated with poorer wellbeing, less is known about the specific psychological process through which this association emerges in everyday financial decision contexts. The present study proposes decision fatigue as one such mechanism. Decision fatigue refers to a state of reduced willingness or capacity to continue effortful decision making after repeated acts of choice, comparison, and judgment (Pignatiello et al., 2020; Polman and Vohs, 2016). In financial life, decision fatigue may arise when people repeatedly evaluate spending, debt, savings, borrowing, investment risks, or future financial trade-offs. For financially insecure individuals, these decisions are not merely technical calculations. They are emotionally and cognitively demanding because the perceived cost of error is high.

The decision fatigue literature is not theoretically uncontested. Earlier limited-resource accounts of self-control emphasized depletion after repeated regulation, whereas subsequent process and motivational models emphasize shifts in attention, perceived effort, value, and motivation (Vohs et al., 2008; Hagger et al., 2010; Inzlicht et al., 2014). The present study does not depend on a strong biological-resource interpretation. Instead, it adopts a broader psychological interpretation: repeated, effortful, and emotionally salient financial choices can produce fatigue, avoidance, lower persistence, and a reduced sense of mental energy (Boksem and Tops, 2008; Müller and Apps, 2019). This process is compatible with research on scarcity and cognitive bandwidth, which suggests that financial concern can consume cognitive resources and increase the subjective burden of everyday decisions (Mani et al., 2013; Haushofer and Fehr, 2014).

This mechanism is also relevant to intertemporal choices. Financial decisions often require a trade-off between present relief and future security, such as whether to save or spend, repay debt or maintain liquidity, borrow now or delay consumption, and accept immediate benefits at the cost of long-term obligations. When decision fatigue accumulates, individuals may have less energy for future-oriented evaluation, may avoid complex long-term planning, and may rely more heavily on short-term relief or default choices. Thus, decision fatigue may affect wellbeing not only directly, by reducing vitality and positive functioning, but also indirectly, by weakening the capacity to sustain intertemporal financial planning under insecurity.

The present study uses an integrated empirical design to examine this framework. The European Social Survey (ESS) analysis tests the general association between economic insecurity and positive wellbeing in a large cross-national sample. It does not include digital finance variables or decision fatigue measures and is therefore used only to evaluate the general H1 association. The three-wave Chinese survey then tests the full mediation model among adult users of digitally mediated financial tools. This division of evidence is central to the manuscript: the cross-national public dataset establishes external validity for the insecurity–wellbeing association, whereas the three-wave survey evaluates the proposed mechanism within the digital financial decision context.

The following hypotheses are tested:

- H1. Economic insecurity is negatively associated with positive wellbeing.
- H2. Economic insecurity is positively associated with decision fatigue in financial decision contexts.
- H3. Decision fatigue is negatively associated with positive wellbeing.
- H4. Decision fatigue mediates the negative association between economic insecurity and positive wellbeing.

The study makes three contributions. First, it clarifies economic insecurity as a subjective psychological stressor rather than merely a structural economic condition. Second, it introduces financial decision fatigue as a mechanism linking insecurity to lower positive wellbeing. Third, it situates this mechanism in digitally mediated financial decision environments while avoiding the assumption that digital finance is inherently harmful. The central argument is more conditional: digital financial tools may intensify decision exposure for people who already feel economically vulnerable.

## 2 Theoretical background and hypothesis development

### 2.1 Economic insecurity as a subjective psychological stressor

Economic insecurity refers to the perceived possibility that one's financial situation may deteriorate and that available resources may be insufficient to cope with future demands. This construct is distinct from income, employment status, or objective wealth. Two individuals with similar resources may differ in whether they feel secure, whether they believe they can absorb unexpected expenses, and whether they perceive their future financial life as controllable (Adler et al., 2000; Singh-Manoux et al., 2003; Smith et al., 2012). This subjective dimension is central because psychological stress depends not only on exposure to hardship but also on appraisal, anticipated loss, and perceived coping capacity.

Prior research shows that financial strain, economic uncertainty, and perceived insecurity are associated with poorer mental health and lower life satisfaction (Rohde et al., 2016; Kopasker et al., 2018; De Witte et al., 2016; Ryu and Fan, 2023). These associations often remain after controlling for objective socioeconomic status, suggesting that subjective vulnerability carries explanatory value beyond material resources alone. Insecurity may keep attention oriented toward threat, generate repeated worry about possible deterioration, and reduce the sense that daily life is predictable and manageable (Haushofer and Fehr, 2014; Mani et al., 2013). Such psychological conditions are likely to weaken positive wellbeing by reducing happiness, vitality, calmness, and favorable life evaluation.

Accordingly, economic insecurity is expected to be negatively associated with positive wellbeing (H1).

### 2.2 Decision fatigue in repeated and complex financial choice contexts

Decision fatigue captures a state in which repeated decisions make subsequent choices feel more effortful, aversive, or difficult to sustain (Pignatiello et al., 2020; Polman and Vohs, 2016). In the present study, the concept is applied specifically to financial decision contexts. Financial choices often require comparison across uncertain alternatives, attention to delayed outcomes, and regulation of fear or regret. These demands become stronger when individuals feel economically insecure, because the perceived consequences of a wrong choice become larger.

Digitally mediated financial environments can amplify this process by increasing the frequency and salience of financial decisions. Mobile interfaces, notifications, rankings, recommendation systems, digital credit offers, and real-time account information can make financial choice more convenient, but also more continuous. Information-overload research suggests that more information and more options do not always improve decision quality; they may also increase cognitive load and fragment attention (Eppler and Mengis, 2004; Arnold et al., 2023; Shahrzadi et al., 2024; Marsh et al., 2024). For economically insecure individuals, this environment may repeatedly reactivate concern and encourage further monitoring, comparison, and reevaluation.

Economic insecurity should therefore be positively associated with decision fatigue. Insecurity makes financial decisions feel consequential and encourages repeated monitoring of current and future financial states. This repeated demand may produce fatigue even when no single decision is objectively large. We therefore expect economic insecurity to be positively associated with financial decision fatigue (H2).

### 2.3 Positive wellbeing as an outcome of sustained decision strain

Positive wellbeing concerns favorable subjective functioning, including happiness, life satisfaction, energy, calmness, and engagement with daily life (Diener et al., 1999; Topp et al., 2015). Sustained decision strain can undermine these indicators in several ways. First, repeated effortful choice can reduce mental energy and increase the subjective sense of exhaustion. Second, fatigue can encourage avoidance and procrastination, which may create dissatisfaction and a diminished sense of control. Third, unresolved financial choices may keep attention fixed on obligations and possible mistakes, leaving less opportunity for restoration and positive affect (Boksem and Tops, 2008; Müller and Apps, 2019).

The link between decision fatigue and wellbeing is also consistent with broader theories of self-regulation. Whether interpreted as resource depletion or as a motivational shift away from continued effort, repeated self-regulatory demand can reduce persistence and increase perceived burden (Hagger et al., 2010; Inzlicht et al., 2014). In financial life, this implies that people who feel worn down by repeated financial comparisons and decisions may report lower wellbeing even if they are not clinically distressed. We therefore expect decision fatigue to be negatively associated with positive wellbeing (H3).

### 2.4 The mediating role of decision fatigue

The preceding arguments imply a mediation process. Economic insecurity encourages vigilance toward future loss and limited coping capacity. In digitally mediated financial decision environments, this vigilance can translate into repeated checking, comparison, and effortful judgment. Over time, these decision demands are associated with greater decision fatigue. Decision fatigue, in turn, is associated with lower positive wellbeing because it reduces energy, calmness, and the sense that everyday life remains manageable (Pignatiello et al., 2020; Polman and Vohs, 2016; Topp et al., 2015).

This account clarifies why the insecurity–wellbeing association should not be treated as purely direct. Economic insecurity may be linked to lower wellbeing not only because financially insecure people have fewer resources, but also because insecurity changes how they must think, monitor, compare, and regulate themselves in everyday financial life. Hence, decision fatigue is expected to mediate the negative association between economic insecurity and positive wellbeing (H4).

## 3 Methods

### 3.1 Overall empirical strategy

The empirical strategy contains two complementary components. The ESS analysis tests whether economic insecurity is associated with lower positive wellbeing in a large and heterogeneous cross-national adult sample. Because the ESS does not measure digital finance use or decision fatigue, this component is used only for H1. The three-wave survey tests H1–H4 among adult users of digitally mediated financial tools. This second component provides temporal ordering between predictor, mediator, and outcome, but it is still interpreted cautiously because the measures are self-reported and the outcome time window partly overlaps with preceding waves.

### 3.2 Public dataset: European Social Survey Round 6

#### 3.2.1 Data, country coverage, and sample construction

The public-data component used the European Social Survey Round 6, fielded in 2012–2013. Round 6 was selected because it contains a rotating module on personal and social wellbeing together with core items on subjective financial strain. The participating countries in the analytic file were Albania, Belgium, Bulgaria, Cyprus, Czech Republic, Denmark, Estonia, Finland, France, Germany, Hungary, Iceland, Ireland, Israel, Italy, Kosovo, Lithuania, Netherlands, Norway, Poland, Portugal, Russian Federation, Slovak Republic, Slovenia, Spain, Sweden, Switzerland, Ukraine, and the United Kingdom. The initial pooled sample contained 54,673 respondents.

Although the ESS target population includes persons aged 15 years and older living in private households, the present analysis was restricted to adult respondents aged 18 years or above. Respondents were retained if they had valid responses on economic insecurity, happiness, life satisfaction, age, gender, education, employment status, marital status, subjective household income position, and self-rated health. Respondents were excluded if they had missing values on any focal variable, refused to answer core items, or gave non-substantive responses such as “don't know” to the main wellbeing or financial-strain variables. The final complete-case sample consisted of 43,782 respondents nested in 29 countries. The mean age was 46.92 years (*SD* = 18.11), 53.4% were women, 56.7% were currently employed, and 61.2% were married or cohabiting.

Post-stratification weights supplied by the ESS were used in the main descriptive analyses and in the primary individual-level regression models to improve within-country representativeness. Multilevel models included country random intercepts to account for respondent nesting within countries, and robust standard errors were reported. Because the primary interest was the individual-level association between perceived financial strain and wellbeing rather than estimating a population-size-weighted European average, population-size weights were not used in the primary multilevel specification. As a sensitivity check, pooled models were re-estimated with combined post-stratification and population-size weights, and the focal association remained substantively unchanged. Additional sensitivity checks used country fixed effects and ordered-response models. Because the primary interest was not country-level institutional explanation, no country-level predictors were added to the baseline model.

#### 3.2.2 Measures

##### 3.2.2.1 Economic insecurity

Economic insecurity was operationalized as subjective difficulty living on present household income. Responses were recoded so that higher values indicated greater perceived economic difficulty (1 = living comfortably, 4 = finding it very difficult). This measure captures perceived financial vulnerability rather than objective income. Because the ESS Round 6 public data do not provide a directly parallel multi-item financial-insecurity scale, this item was retained as a single-item indicator of perceived household income strain. To avoid introducing a non-replicable composite, robustness analyses treated the item both as a continuous ordinal score and as an ordered categorical predictor; the substantive conclusion was unchanged.

##### 3.2.2.2 Positive wellbeing

Positive wellbeing in the ESS was measured with happiness and life satisfaction, both scored from 0 to 10. The two items were averaged to form a subjective wellbeing composite. Their correlation was strong (*r* = 0.73, *p* < 0.001), supporting combined use. Additional models examined life satisfaction and happiness separately.

##### 3.2.2.3 Control variables

Controls included age, gender, years of education, employment status, marital status, subjective household income position, and self-rated health. Because subjective income position and self-rated health may be conceptually close to perceived insecurity and wellbeing, additional sensitivity models were estimated with these controls removed separately and jointly.

### 3.3 Three-wave survey of adult digital finance users

#### 3.3.1 Participants and procedure

The mechanism-testing component used a three-wave online survey of adults in China who had used at least one digitally mediated financial tool during the previous 6 months. Eligible tools included mobile banking, online budgeting tools, digital credit products, online investment platforms, robo-advisory services, and recommendation-supported financial applications. Participants were recruited through the Credamo online research panel, which maintains verified adult respondents and allows quota monitoring by gender and age group. Fieldwork was conducted in three 1-week windows: T1 from April 15 to April 21, 2024, T2 from April 22 to April 28, 2024, and T3 from April 29 to May 5, 2024. T1 measured economic insecurity, demographic characteristics, financial background, and digital finance tool use; T2 measured financial decision fatigue and validation constructs; T3 measured positive wellbeing and supplementary robustness items.

Participants received platform-administered monetary compensation of RMB 5 after T1, RMB 6 after T2, RMB 8 after T3, and an additional RMB 5 completion bonus after finishing all three waves. Quality-control procedures were specified before analysis. Respondents were removed if they failed either of two attention-check items, gave identical responses to all items in a multi-item block, completed a wave below the wave-specific minimum completion-time threshold, reported inconsistent demographic information across waves, or failed to provide a valid matched identifier. The minimum completion-time screen followed the pre-specified rule of excluding responses completed in less than one third of the observed median completion time for the corresponding wave. The observed median completion times were 423 s at T1, 312 s at T2, and 261 s at T3, yielding exclusion thresholds of less than 141 s, less than 104 s, and less than 87 s, respectively. These exact thresholds are reported in Table 1 to improve reproducibility. Straight-lining was defined as providing the same response option to all items in a multi-item scale after reverse-keyed or attention-check items had been excluded. A total of 2,184 respondents completed T1, 1,786 completed T2, and 1,594 completed T3. Among the 1,594 respondents who reached T3, 82 were excluded after applying the pre-specified quality-control screen because of failed attention checks, straight-lining, implausibly short completion time, inconsistent demographic information, or invalid matched identifiers. These exclusion categories were not treated as mutually exclusive because a small number of respondents met more than one quality-control criterion. The final matched sample contained 1,512 respondents, corresponding to a retention rate of 69.2% from T1 and 94.9% from T3 completion.

**Table 1:** Minimum completion-time thresholds used in the three-wave survey.

Wave	Median completion time	Threshold rule	Minimum threshold
T1	423 s	<1/3 of median	<141 s
T2	312 s	<1/3 of median	<104 s
T3	261 s	<1/3 of median	<87 s

Thresholds were calculated separately for each wave from the observed median completion time before applying the quality-control exclusion screen. Respondents below the relevant wave-specific threshold were flagged for implausibly short completion time.

The final sample had a mean age of 31.84 years (*SD* = 8.74); 52.1% were women; 76.5% held a bachelor's degree or above; 80.2% were in full-time employment, 11.7% were self-employed or flexibly employed, and 8.1% were not currently employed. Regarding digital finance use, 64.7% reported weekly use, 24.9% monthly use, and 10.4% occasional use. Attrition analysis indicated that retained and dropped participants did not differ significantly in T1 economic insecurity, gender, education, or income band (all *p*>0.10). Dropped participants were slightly younger than retained participants (*d* = 0.10), but this difference was small. Age was included as a control variable in all structural models. Table 2 summarizes the retention process and quality-control exclusions.

**Table 2:** Three-wave survey retention and quality-control flow.

Stage	Respondents	Retention/exclusion information
Completed T1	2,184	Baseline eligible adult digital finance users
Completed T2	1,786	81.8% of T1 respondents
Completed T3	1,594	73.0% of T1 respondents
Excluded after T3 screening	82	Failed attention checks, straight-lining, short duration, or identifier inconsistency
Final matched analytic sample	1,512	69.2% of T1; 94.9% of T3 completers

Quality-control rules were specified before analysis. Straight-lining was evaluated within multi-item blocks after excluding attention-check items. The 82 exclusions are reported as the total number removed after applying the full screening procedure; category-specific counts are not summed because some exclusion criteria could overlap.

#### 3.3.2 Measures

##### 3.3.2.1 Economic insecurity (T1)

Economic insecurity was measured with six items capturing subjective financial vulnerability. Participants rated each item from 1 = *strongly disagree* to 7 = *strongly agree*. The items were: “I worry that my financial situation may deteriorate in the near future,” “Unexpected financial expenses would be difficult for me to manage,” “I am uncertain whether my income will remain sufficient,” “Small financial mistakes could seriously affect my daily life,” “I often reconsider spending because my financial future feels uncertain,” and “I feel vulnerable to sudden changes in my financial circumstances.” The scale showed high internal consistency (α = 0.90, ω = 0.91).

##### 3.3.2.2 Decision fatigue (T2)

Financial decision fatigue was measured with six items adapted to financial decision contexts from conceptual work on decision fatigue and self-regulatory strain. Responses ranged from 1 = *strongly disagree* to 7 = *strongly agree*. The items were: “After making several financial decisions, I feel mentally drained,” “I postpone financial choices because I feel exhausted,” “Repeated financial comparisons make it hard for me to keep thinking clearly,” “I feel too tired to evaluate financial options carefully,” “After checking financial information, I prefer not to make more decisions,” and “Financial decisions require more mental effort than I can easily maintain.” Reliability was high (α = 0.92, ω = 0.92).

##### 3.3.2.3 Positive wellbeing (T3)

Positive wellbeing was assessed with the WHO-5 Wellbeing index. Participants reported how often they had experienced five positive states during the previous 2 weeks on a 0–5 scale. Scores were summed and multiplied by 4 to create the standard 0–100 WHO-5 score; item-mean scores are also reported for interpretability. Reliability was strong (α = 0.89, ω = 0.90). Because the WHO-5 refers to the previous 2 weeks and the study waves were separated by approximately 1 week, the T3 recall window partly overlapped with the earlier measurement period. This issue is explicitly addressed in the interpretation and limitations.

##### 3.3.2.4 Supplementary validation constructs

To evaluate convergent and discriminant validity, the survey included short measures of financial stress, perceived financial wellbeing, perceived financial control, and general anxiety. Financial stress captured worry and pressure related to money management. Perceived financial wellbeing captured the extent to which respondents felt able to meet current obligations and maintain future financial security. Perceived financial control assessed whether respondents felt capable of regulating financial choices. General anxiety was included to test whether financial decision fatigue was distinguishable from a broader distress state. These constructs were used only for measurement validation and robustness checks, not as primary outcomes.

##### 3.3.2.5 Control variables

Survey models controlled for age, gender, educational attainment, employment status, monthly income band, debt burden, self-rated financial literacy, and frequency of digitally mediated financial tool use. Because debt burden and financial literacy may function as contextual or mechanistic variables rather than pure confounders, sensitivity analyses estimated models with and without these controls.

### 3.4 Analytical strategy

All analyses were conducted in R 4.3.2 (R Foundation for Statistical Computing, Vienna, Austria) using the survey, lme4, lavaan, and semTools packages for survey-weighted regression, multilevel modeling, confirmatory factor analysis, structural equation modeling, bootstrap inference, and measurement invariance testing. The ESS analysis used post-stratification weights for descriptive estimates and weighted regression checks. Multilevel linear models included country random intercepts and robust standard errors. Intraclass correlation coefficients were calculated from unconditional models, and pseudo-*R*^2^ values were defined as the proportional reduction in residual variance relative to the corresponding baseline model. The baseline model was specified as Equation 1:


$$\begin{array}{c} \text{PW}{\text{B}}_{ij}=\gamma_{00}+\gamma_{10}\text{E}{\text{I}}_{ij}+\underset{k}{\sum }\gamma_{k0}\text{Control}{\text{s}}_{kij}+u_{0j}+r_{ij}, \end{array}$$ (1)

where PWB_*ij*_ denotes positive wellbeing for respondent *i* in country *j*, EI_*ij*_ denotes economic insecurity, *u*_0*j*_ denotes the country-level random intercept, and *r*_*ij*_ denotes the individual-level residual.

The three-wave survey analysis proceeded in six steps. First, descriptive statistics, zero-order correlations, and attrition diagnostics were examined. Second, item-level diagnostics were evaluated using corrected item-total correlations, standardized factor loadings, skewness, and kurtosis. Third, the sample was randomly split into two halves for cross-validation: exploratory factor analysis was conducted in the first half and confirmatory factor analysis was conducted in the second half before the full-sample CFA was estimated. Fourth, structural equation modeling tested the mediation model using 5,000 bias-corrected bootstrap resamples. Fifth, moderation and heterogeneity were evaluated using interaction terms, multi-group SEM with equality constraints, and bootstrapped differences in conditional indirect effects. Sixth, robustness checks assessed alternative operationalizations, possible overcontrol, common-method variance, and reverse-order specifications. Missing values within retained cases were handled using full-information maximum likelihood in SEM. Non-normality was addressed using robust maximum likelihood estimation. Multiple heterogeneity tests were interpreted cautiously, and the Benjamini–Hochberg procedure was used as a supplementary false-discovery-rate check. The mediation equations were specified as Equations 2 and 3:


$$\begin{array}{cc} \text{D}{\text{F}}_{i} & =a_{0}+a_{1}\text{E}{\text{I}}_{i}+\underset{k}{\sum }a_{k}\text{Control}{\text{s}}_{ki}+\varepsilon_{1i}, \end{array}$$ (2)

$$\begin{array}{cc} \text{PW}{\text{B}}_{i} & =b_{0}+c^{\prime }\text{E}{\text{I}}_{i}+b_{1}\text{D}{\text{F}}_{i}+\underset{k}{\sum }b_{k}\text{Control}{\text{s}}_{ki}+\varepsilon_{2i}. \end{array}$$ (3)

## 4 Results

### 4.1 Descriptive statistics and preliminary evidence

Table 3 reports descriptive statistics and zero-order correlations for the focal variables. In the ESS sample, economic insecurity was negatively correlated with positive wellbeing (*r* = −0.34, *p* < 0.001). In the three-wave survey, economic insecurity was positively correlated with decision fatigue (*r* = 0.49, *p* < 0.001), economic insecurity was negatively correlated with positive wellbeing (*r* = −0.41, *p* < 0.001), and decision fatigue was negatively correlated with positive wellbeing (*r* = −0.44, *p* < 0.001). These bivariate patterns were consistent with the hypothesized directions, although formal hypothesis testing relied on the multivariate models below.

**Table 3:** Descriptive statistics and zero-order correlations of focal variables.

Dataset	Variable	Mean	SD	1	2	3
ESS (*N* = 43,782)	1. Economic insecurity	2.31	0.87	1		
ESS	2. Positive wellbeing	6.74	1.86	−0.34	1	
Three-wave (*N* = 1,512)	1. Economic insecurity	4.34	1.08	1		
Three-wave	2. Decision fatigue	4.02	1.12	0.49	1	
Three-wave	3. Positive wellbeing	3.18	0.91	−0.41	−0.44	1

All correlations are significant at *p* < 0.001. Positive wellbeing in the three-wave survey is reported on the WHO-5 item-mean metric (0–5); the corresponding standard WHO-5 score is approximately mean × 20.

**Figure 1:**
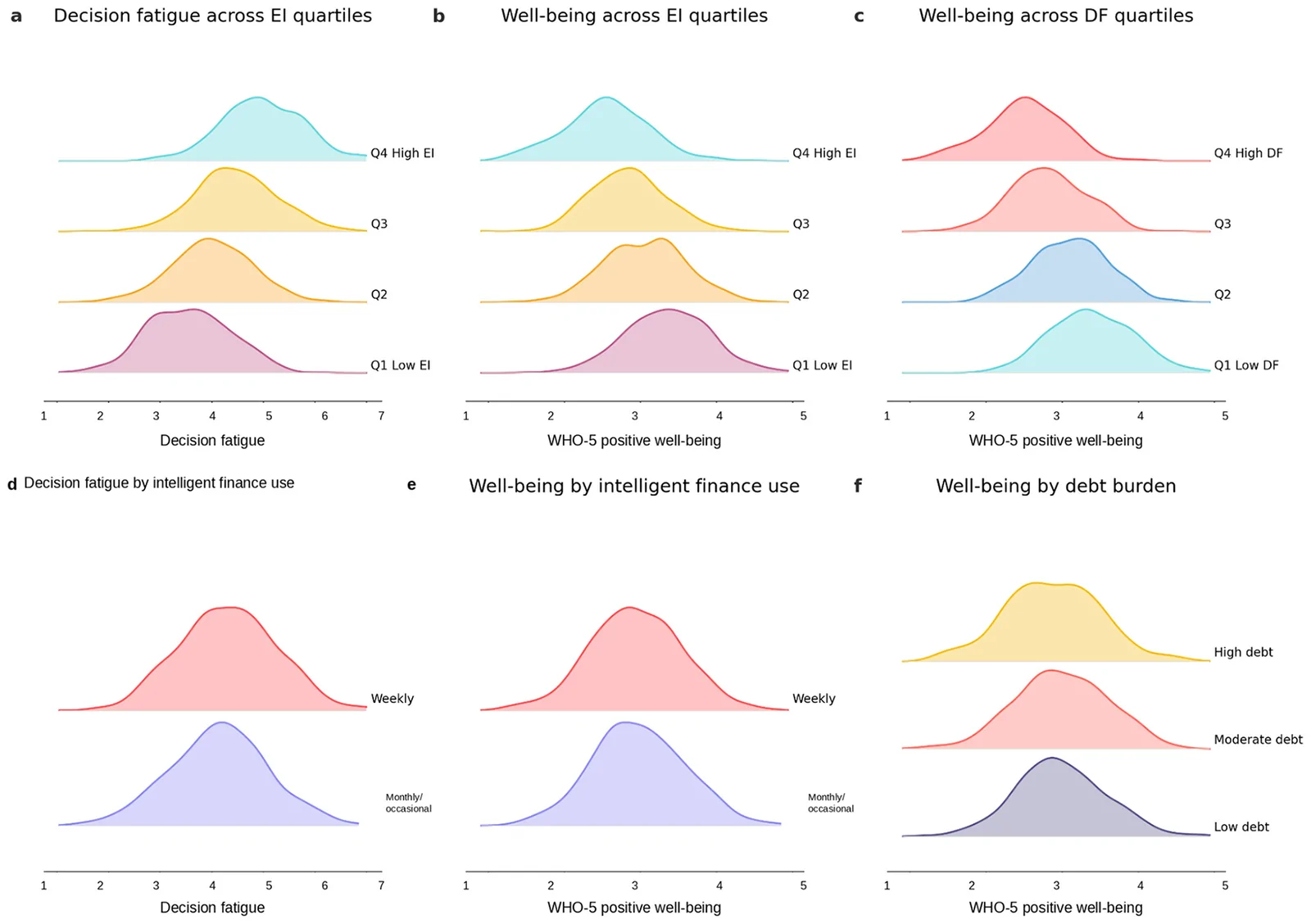
Distributional gradients of economic insecurity, decision fatigue, and WHO-5 positive wellbeing. **(a–c)** Show shifts in decision fatigue and WHO-5 positive wellbeing across quartiles of economic insecurity and decision fatigue. **(d–f)** Compare distributions across fully written-out digital finance tool-use groups (weekly use, monthly use, and occasional use) and debt-burden groups.

Figure 1 shows that higher economic insecurity corresponded to a rightward shift in decision fatigue and a leftward shift in positive wellbeing. Similar distributional changes appeared across digital finance use and debt burden groups. These patterns are descriptive and are not interpreted as causal evidence, but they provide an intuitive visual basis for the multivariate tests.

### 4.2 Public-data evidence for H1

An unconditional random-intercept model indicated that 6.2% of the variance in positive wellbeing was between countries, supporting the use of a multilevel specification. Table 4 summarizes the main ESS results. Economic insecurity was negatively associated with positive wellbeing after demographic and socioeconomic controls were included [*b* = −0.56, *SE* = 0.02, 95% CI [−0.60, −0.52], *p* < 0.001]. The coefficient remained stable when country fixed effects were used [*b* = −0.49, *SE* = 0.02, 95% CI [−0.53, −0.45], *p* < 0.001] and when life satisfaction was examined as a separate outcome [*b* = −0.59, *SE* = 0.02, 95% CI [−0.63, −0.55], *p* < 0.001]. These findings support H1 in the cross-national public dataset.

**Table 4:** Main-effect results based on the ESS public dataset.

Model	Specification	*b*	95% CI	*p*
Model 1	Controls only	–	–	–
Model 2	Random-intercept model	–0.56	[−0.60, −0.52]	<0.001
Model 3	Country fixed effects	–0.49	[−0.53, −0.45]	<0.001
Model 4	Life satisfaction outcome	–0.59	[−0.63, −0.55]	<0.001
Model 5	Happiness outcome	–0.52	[−0.56, −0.48]	<0.001

*N* = 43, 782. Coefficients represent the association between economic insecurity and positive wellbeing. Models include age, gender, education, employment status, marital status, subjective household income position, and self-rated health unless otherwise stated. Post-stratification weights and robust standard errors were used.

**Figure 2:**
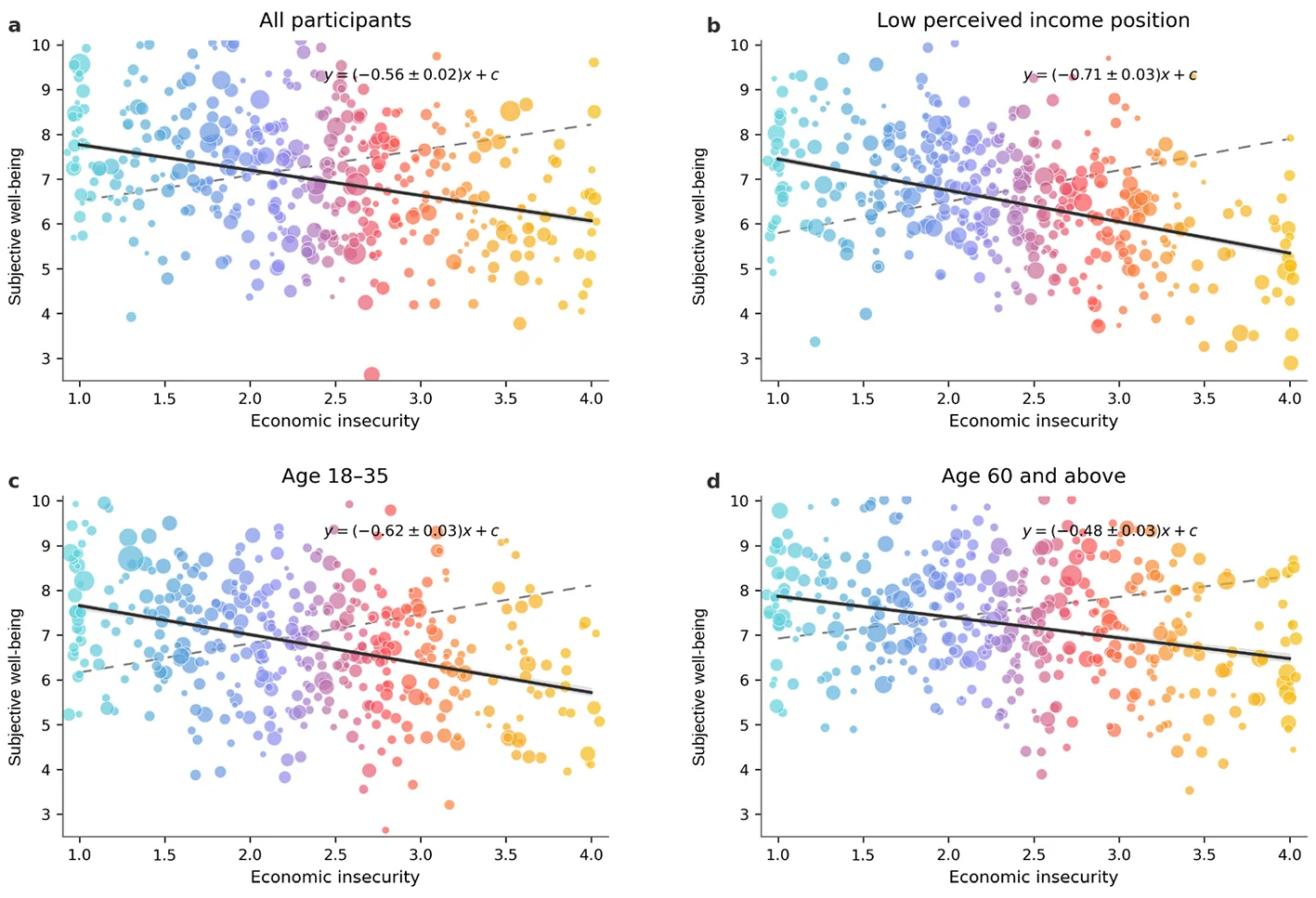
Adjusted association between economic insecurity and subjective wellbeing in the ESS public dataset. **(a)** All participants. **(b)** Respondents with low perceived income position. **(c)** Respondents aged 18-35. **(d)** Respondents aged 60 and above. Each panel visualizes predicted subjective wellbeing levels across economic insecurity after covariate adjustment. Error bands represent 95% confidence intervals.

Figure 2 illustrates that the negative association was continuous across the observed range of economic insecurity. The gradient was steeper among respondents with lower perceived income position and among younger adults, suggesting that the association is stronger in contexts of greater perceived vulnerability. These patterns were examined formally in the interaction analyses below.

### 4.3 Subgroup and interaction analyses in the ESS dataset

Subgroup analyses were conducted by perceived income position and age group. The negative association between economic insecurity and positive wellbeing was strongest among respondents with low perceived income position (*b* = −0.71, *SE* = 0.03, *p* < 0.001), moderate among those with middle perceived income position (*b* = −0.55, *SE* = 0.03, *p* < 0.001), and smaller among those with high perceived income position (*b* = −0.39, *SE* = 0.03, *p* < 0.001). A formal interaction model confirmed that perceived income position significantly moderated the association [*b*_EI × income_ = 0.08, *SE* = 0.01, 95% CI [0.06, .10], *p* < 0.001], indicating that the negative wellbeing association was weaker at higher perceived income positions. Table 5 summarizes the subgroup slopes and formal interaction tests in the ESS dataset.

**Table 5:** Subgroup slopes and formal interaction tests in the ESS dataset.

Group/moderator	*n*	Slope *b*	95% CI	*p*
Low perceived income position	14,206	–0.71	[−0.77, −0.65]	<0.001
Middle perceived income position	18,347	–0.55	[−0.61, −0.49]	<0.001
High perceived income position	11,229	–0.39	[−0.45, −0.33]	<0.001
EI × income position	43,782	0.08	[0.06, 0.10]	<0.001
Age 18–35	11,564	–0.62	[−0.68, −0.56]	<0.001
Age 36–59	17,823	–0.56	[−0.62, −0.50]	<0.001
Age 60 and above	14,395	–0.48	[−0.54, −0.42]	<0.001
EI × age	43,782	0.004	[0.002, 0.006]	<0.001

Age-group results showed a similar pattern. The coefficient was somewhat larger among younger adults aged 18–35 (*b* = −0.62, *SE* = 0.03, *p* < 0.001) than among middle-aged adults (*b* = −0.56, *SE* = 0.03, *p* < 0.001) and older adults (*b* = −0.48, *SE* = 0.03, *p* < 0.001). A continuous interaction between economic insecurity and age was significant but small [*b*_EI × age_ = 0.004, *SE* =.001, 95% CI [.002, .006], *p* < 0.001]. These findings are interpreted as evidence of moderation, but the effect sizes indicate that the main association is broadly present across groups.

### 4.4 Measurement validation in the three-wave survey

Before testing the mediation model, the psychometric properties of the focal constructs were evaluated at both item and construct levels. Item distributions did not indicate severe non-normality: absolute skewness values were below 1.20 and absolute kurtosis values were below 1.50 for all focal items. Corrected item-total correlations were all above 0.60, and standardized factor loadings were all above 0.70. These results supported retention of all items. Table 6 reports the item-level diagnostics.

**Table 6:** Item-level diagnostics for focal constructs in the three-wave survey.

Construct/item	Mean	SD	Loading	Item-total *r*	Skew/Kurtosis
EI1: worry about financial deterioration	4.51	1.33	0.80	0.74	0.18/–0.71
EI2: difficulty managing unexpected expenses	4.63	1.29	0.83	0.76	0.11/–0.63
EI3: uncertainty about income sufficiency	4.28	1.36	0.78	0.71	0.24/–0.69
EI4: consequences of small financial mistakes	4.19	1.41	0.73	0.66	0.31/–0.78
EI5: reconsidering spending due to uncertainty	4.38	1.32	0.75	0.69	0.20/–0.65
EI6: vulnerability to sudden financial changes	4.06	1.39	0.70	0.63	0.36/–0.74
DF1: mentally drained after financial decisions	4.17	1.34	0.84	0.77	0.27/–0.61
DF2: postponing financial choices due to exhaustion	3.94	1.41	0.78	0.70	0.39/–0.70
DF3: repeated comparisons reduce clarity	4.08	1.36	0.86	0.78	0.32/–0.65
DF4: too tired to evaluate options carefully	3.89	1.43	0.81	0.73	0.43/–0.73
DF5: avoiding more decisions after checking information	3.97	1.38	0.76	0.69	0.35/–0.68
DF6: financial decisions require unsustainable effort	4.05	1.35	0.72	0.66	0.30/–0.64
WHO1: cheerful and in good spirits	3.26	0.97	0.79	0.72	–0.25/–0.51
WHO2: calm and relaxed	3.08	1.02	0.76	0.69	–0.13/–0.57
WHO3: active and vigorous	3.11	1.04	0.84	0.77	–0.17/–0.49
WHO4: woke up fresh and rested	3.01	1.08	0.71	0.64	–0.09/–0.61
WHO5: daily life filled with interest	3.44	0.98	0.80	0.73	–0.31/–0.43

EI, economic insecurity; DF, decision fatigue; WHO, WHO-5 positive wellbeing item. Loadings are standardized estimates from the full-sample confirmatory factor model.

Reliability and convergent validity were adequate for economic insecurity (α = 0.90, ω = 0.91, CR = 0.91, AVE = 0.62), decision fatigue (α = 0.92, ω = 0.92, CR = 0.92, AVE = 0.65), and WHO-5 positive wellbeing (α = 0.89, ω = 0.90, CR = 0.90, AVE = 0.64). Standardized factor loadings ranged from 0.70 to 0.83 for economic insecurity, 0.72 to 0.86 for decision fatigue, and 0.71 to 0.84 for positive wellbeing. Corrected item-total correlations ranged from 0.63 to 0.78, supporting item consistency. Table 7 summarizes reliability, convergent validity, and item-diagnostic ranges for the three focal constructs.

**Table 7:** Reliability, convergent validity, and item diagnostics for the three-wave survey constructs.

Construct	α	ω	CR	AVE	Loading range	Item-total range
Economic insecurity	0.90	0.91	0.91	0.62	0.70–0.83	0.63–0.76
Decision fatigue	0.92	0.92	0.92	0.65	0.72–0.86	0.66–0.78
Positive wellbeing	0.89	0.90	0.90	0.64	0.71–0.84	0.64–0.77

A split-sample validation was also conducted to reduce dependence on a single measurement model. In the exploratory half-sample (*n* = 756), parallel analysis supported a three-factor solution corresponding to economic insecurity, decision fatigue, and positive wellbeing. All primary loadings exceeded 0.68 and cross-loadings were below 0.25. In the confirmatory half-sample (*n* = 756), the three-factor CFA showed acceptable fit (CFI = 0.952, TLI = 0.944, RMSEA = 0.045, SRMR = 0.043). The full-sample CFA was then estimated for the structural analyses. Confirmatory factor analysis supported the hypothesized three-factor structure: χ^2^(167) = 486.37, CFI = 0.958, TLI = 0.950, RMSEA = 0.042, SRMR = 0.039. All loadings were statistically significant at *p* < 0.001. A two-factor model combining economic insecurity and decision fatigue fit worse, χ^2^(169) = 941.62, CFI = 0.895, TLI = 0.881, RMSEA = 0.068, SRMR = 0.074. A one-factor model fit poorly, χ^2^(170) = 1876.44, CFI = 0.771, TLI = 0.742, RMSEA = 0.101, SRMR = 0.111. These comparisons supported discriminant validity. Additional checks showed that the square root of AVE for each construct exceeded its correlations with the other constructs, further supporting discriminant validity.

Measurement invariance was examined across high vs. low-to-moderate digital finance use and high vs. low-to-moderate debt burden. Configural and metric invariance were supported in both comparisons (all ΔCFI <0.010 and ΔRMSEA <0.015), indicating that group comparisons of structural paths were interpretable. Scalar invariance was acceptable for digital finance use and partially supported for debt burden after freeing one item intercept. Because the focal heterogeneity tests concern path differences rather than mean comparisons, the supported configural and metric invariance provided an adequate basis for multi-group SEM.

Convergent and discriminant validity were further evaluated against supplementary constructs. Decision fatigue was positively correlated with financial stress (*r* = 0.58, *p* < 0.001) and general anxiety (*r* = 0.43, *p* < 0.001), and negatively correlated with perceived financial control (*r* = −0.46, *p* < 0.001) and financial wellbeing (*r* = −0.40, *p* < 0.001). The correlations were theoretically consistent but did not approach redundancy, supporting the interpretation that financial decision fatigue overlaps with distress and control perceptions while remaining empirically distinct from them. Table 8 reports these correlations.

**Table 8:** Associations between focal constructs and supplementary validation variables.

Supplementary construct	Economic insecurity	Decision fatigue	Positive wellbeing
Financial stress	0.62	0.58	–0.45
Financial wellbeing	–0.55	–0.40	0.52
Perceived financial control	–0.48	–0.46	0.38
General anxiety	0.39	0.43	–0.49

All correlations are significant at *p* < 0.001. The pattern supports convergent validity while indicating that decision fatigue is not reducible to general anxiety or financial stress.

### 4.5 Main mediation results from the three-wave survey

The structural equation model fit the data well: χ^2^(184) = 538.71, CFI = 0.955, TLI = 0.948, RMSEA = 0.043, SRMR = 0.044. As shown in Table 9, T1 economic insecurity positively predicted T2 decision fatigue [*B* = 0.52, *SE* = 0.03, β = 0.50, 95% CI [0.46, 0.58], *p* < 0.001], supporting H2. T2 decision fatigue negatively predicted T3 positive wellbeing [*B* = −0.27, *SE* = 0.03, β = −0.33, 95% CI [−0.33, −0.21], *p* < 0.001], supporting H3. Economic insecurity retained a direct association with positive wellbeing [*B* = −0.17, *SE* = 0.03, β = −0.21, 95% CI [−0.23, −0.11], *p* < 0.001], indicating partial mediation.

**Table 9:** Main mediation results from the three-wave survey.

Path	*B*	*SE*	β	95% CI	*p*
Economic insecurity → Decision fatigue	0.52	0.03	0.50	[0.46, 0.58]	<0.001
Decision fatigue → Positive wellbeing	–0.27	0.03	–0.33	[−0.33, −0.21]	<0.001
Economic insecurity → Positive wellbeing	–0.17	0.03	–0.21	[−0.23, −0.11]	<0.001
Indirect effect	–0.14	–	–	[−0.17, −0.11]	–
Total effect	–0.31	0.03	–0.38	[−0.37, −0.25]	<0.001
*R*^2^ for decision fatigue	0.314				
*R*^2^ for positive wellbeing	0.362				

The indirect effect through decision fatigue was significant [*B* = −0.14, bootstrapped 95% CI [−0.17, −0.11]], supporting H4. The total effect was *B* = −0.31 [*SE* = 0.03, β = −0.38, 95% CI [−0.37, −0.25], *p* < 0.001], and the indirect-to-total ratio was approximately 45%. The model explained 31.4% of the variance in decision fatigue and 36.2% of the variance in positive wellbeing. Because the data are observational and self-reported, these findings are interpreted as temporally ordered associations rather than definitive causal effects.

**Figure 3:**
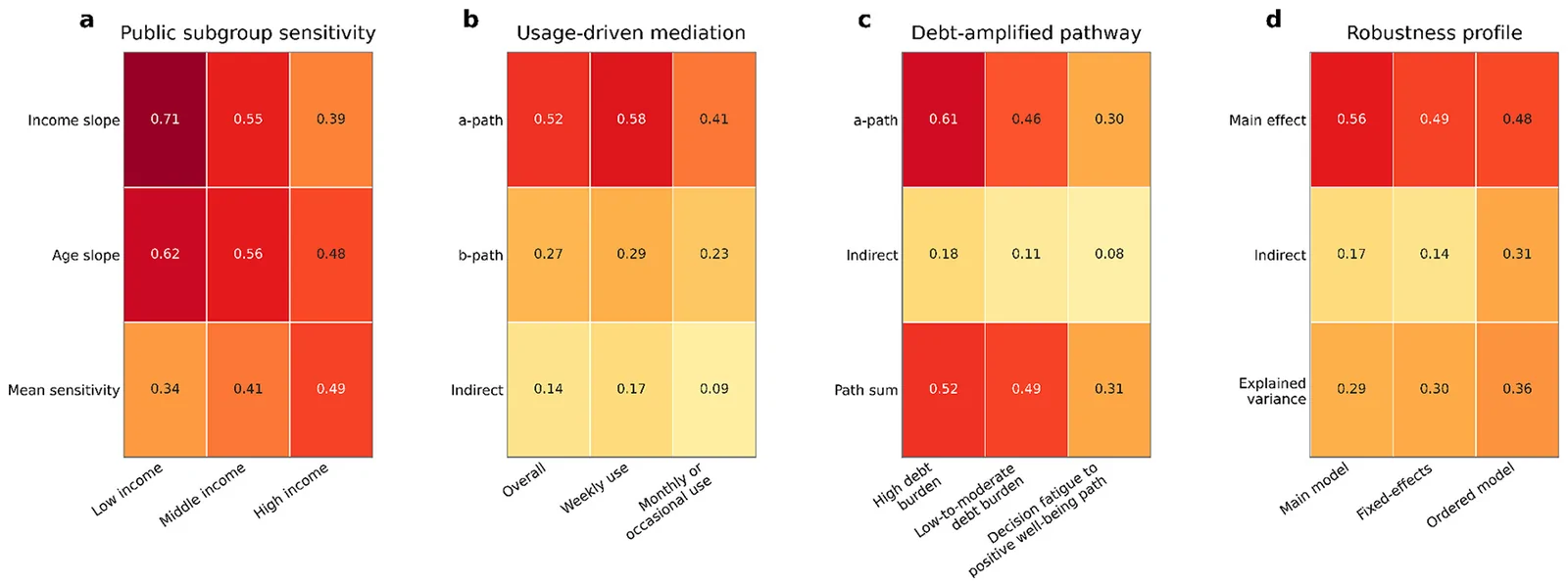
Pathway magnitudes and explanatory power in the three-wave survey. **(a)** Compares the economic insecurity to decision fatigue path across the overall sample and key subgroups. **(b)** Summarizes the decision fatigue to WHO-5 positive wellbeing path. **(c)** Presents the bootstrapped indirect effect. **(d)** Reports explained variance in decision fatigue and WHO-5 positive wellbeing. Error bars represent 95% confidence intervals where applicable.

Figure 3 summarizes the magnitude pattern of the mediation model. The figure highlights that the indirect pathway was not only statistically significant but also substantively meaningful, with a relatively strong path from insecurity to fatigue and a consistent negative path from fatigue to positive wellbeing.

### 4.6 Formal moderation and heterogeneity analyses

#### 4.6.1 Digital finance tool use

To evaluate whether the mediation mechanism was stronger in more decision-intensive digital finance contexts, digital finance tool use was tested as a moderator. Participants using digital finance tools at least weekly were classified as high-frequency users (*n* = 978), and the remaining participants were classified as low-to-moderate users (*n* = 534). The interaction between economic insecurity and digital finance use predicting decision fatigue was significant [*B* = 0.13, *SE* = 0.04, 95% CI [0.05, 0.21], *p* = 0.002]. Multi-group SEM with equality constraints also showed that constraining the EI → DF path to equality across groups worsened model fit [Δχ^2^(1) = 12.84, *p* < 0.001].

The conditional indirect effect was larger among high-frequency users [*B* = −0.17, 95% CI [−0.21, −0.13]] than among low-to-moderate users [*B* = −0.09, 95% CI [−0.13, −0.06]]. The bootstrapped difference in indirect effects was significant [Δ*B* = −0.08, 95% CI [−0.12, −0.04]]. This supports the interpretation that frequent digital finance use strengthens the decision-fatigue pathway, although the finding should be understood as moderation rather than proof that digital tools cause fatigue. Table 10 summarizes the conditional indirect effects and moderation tests in the three-wave survey

**Table 10:** Conditional indirect effects and moderation tests in the three-wave survey.

Moderator/group	*n*	EI → DF	DF → PWB	Indirect effect	Difference
High digital finance usage	978	0.58 (0.04)	–0.29 (0.04)	−0.17 [−0.21, −0.13]	−0.08 [−0.12, −0.04]
Low-to-moderate usage	534	0.41 (0.05)	–0.23 (0.05)	−0.09 [−0.13, −0.06]	–
High debt burden	468	0.61 (0.05)	–0.30 (0.05)	−0.18 [−0.23, −0.13]	−0.07 [−0.12, −0.03]
Low-to-moderate debt burden	1,044	0.46 (0.04)	–0.25 (0.03)	−0.11 [−0.14, −0.08]	–

Values in parentheses are standard errors. Values in brackets are bootstrapped 95% confidence intervals. PWB, positive wellbeing; EI, economic insecurity; DF, decision fatigue. “Difference” refers to the between-group difference in the bootstrapped indirect effect, using the low-to-moderate group as the reference category.

#### 4.6.2 Debt burden

Debt burden was also tested as a moderator because heavier debt obligations may increase the perceived cost of financial mistakes. Participants in the upper tercile of the debt-burden scale were classified as high-debt-burden respondents (*n* = 468), and the remaining respondents formed the low-to-moderate group (*n* = 1, 044). The interaction between economic insecurity and debt burden predicting decision fatigue was significant [*B* = 0.15, *SE* = 0.04, 95% CI [0.07, 0.23], *p* < 0.001]. Equality constraints in multi-group SEM again worsened fit for the EI → DF path [Δχ^2^(1) = 15.21, *p* < 0.001].

The indirect effect was larger among participants with heavier debt burden [*B* = −0.18, 95% CI [−0.23, −0.13]] than among participants with lower-to-moderate debt burden [*B* = −0.11, 95% CI [−0.14, −0.08]]. The difference in indirect effects was significant [Δ*B* = −0.07, 95% CI [−0.12, −0.03]]. These findings suggest that the proposed pathway is stronger when financial obligations are heavier and perceived room for error is smaller. The focal interaction tests for digital finance use and debt burden remained significant after Benjamini–Hochberg correction for the set of heterogeneity tests reported in this section.

**Figure 4:**
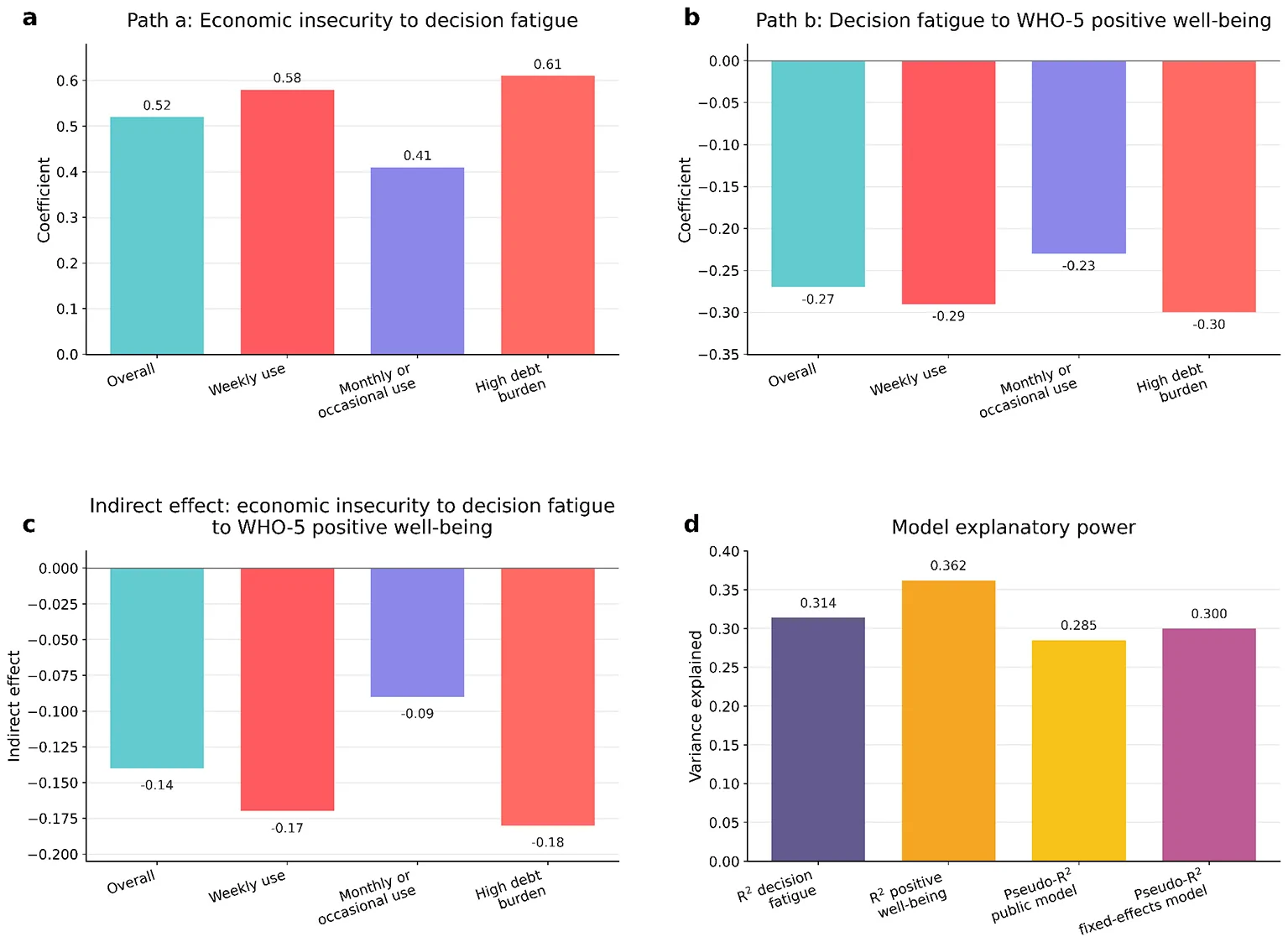
Annotated heatmaps of subgroup sensitivity and mediation strength. **(a)** Summarizes subgroup sensitivity in the ESS dataset. **(b)** Visualizes conditional mediation across digital finance tool-use intensity. **(c)** Presents conditional mediation across debt burden. **(d)** Summarizes robustness checks. Color intensity represents standardized effect magnitude, and annotations report the corresponding coefficient or bootstrapped confidence interval. High digital finance use refers to weekly use, and low-to-moderate use refers to monthly or occasional use.

Figure 4 integrates the subgroup and conditional mediation evidence. The figure is used as a synthesis rather than as a substitute for formal tests. The annotated cells show that stronger mediation occurs when digital finance use is more frequent and when debt burden is heavier, while the robustness panel shows that the core pathway remains directionally stable across model specifications.

### 4.7 Robustness checks

Several robustness analyses were conducted. First, the three-wave dependent variable was modeled using a supplementary life-satisfaction-based positive functioning measure at T3. The indirect effect remained significant [*B* = −0.16, 95% CI [−0.20, −0.12]]. Second, the economic insecurity scale was re-estimated after removing the item most closely tied to immediate expense anxiety; the indirect effect remained significant [*B* = −0.13, 95% CI [−0.16, −0.10]]. Third, the ESS analysis was re-estimated using ordered-response models for happiness and life satisfaction. The odds ratio associated with economic insecurity was 0.62 [95% CI [0.59, 0.65], *p* < 0.001], indicating lower odds of reporting higher wellbeing categories.

Fourth, the ESS model was re-estimated without subjective household income position and self-rated health. Removing subjective income position slightly increased the focal coefficient (*b* = −0.63, *SE* = 0.02, *p* < 0.001), whereas removing self-rated health yielded a similar coefficient (*b* = −0.58, *SE* = 0.02, *p* < 0.001). Removing both controls produced *b* = −0.66 (*SE* = 0.02, *p* < 0.001). These results indicate that the main association was not an artifact of overcontrol, although subjective income and health clearly explain additional variance in wellbeing.

Fifth, the three-wave survey model was re-estimated without debt burden and financial literacy controls. The indirect effect remained significant [*B* = −0.15, 95% CI [−0.18, −0.12]], indicating that the mediation pathway was not dependent on treating these variables as confounders. Sixth, common-method variance was evaluated using the temporal separation of focal measures, an unrotated single-factor diagnostic, and an unmeasured latent method factor. The first factor accounted for 29.8% of variance, and the focal paths remained significant after including the method factor [EI → DF: *B* = 0.49, *p* < 0.001; DF → PWB: *B* = −0.25, *p* < 0.001; indirect effect *B* = −0.12, 95% CI [−0.15, −0.09]].

Finally, reverse-order and cross-lagged specifications were examined. A reverse-order SEM specifying lower positive wellbeing as the predictor of later decision fatigue fit the data less well than the hypothesized model (CFI = 0.923, TLI = 0.912, RMSEA = 0.057, SRMR = 0.060). A cross-lagged specification using T1 economic insecurity, T2 decision fatigue, and T3 positive wellbeing showed that the T1 EI → T2 DF path (β = 0.42, *p* < 0.001) was stronger than the reverse-direction analog (β = 0.11, *p* < 0.01). These analyses support the temporal plausibility of the proposed model, while not eliminating the possibility of reciprocal relationships. Table 11 summarizes the robustness evidence.

**Table 11:** Summary of robustness and sensitivity analyses.

Robustness check	Key estimate	95% CI/fit	Interpretation
Alternative T3 life-satisfaction outcome	Indirect effect = −0.16	[−0.20, −0.12]	Mediation remains significant
Economic insecurity item removed	Indirect effect = −0.13	[−0.16, −0.10]	Not driven by one insecurity item
ESS ordered-response model	OR = 0.62	[0.59, 0.65]	Higher insecurity predicts lower wellbeing category
ESS without subjective income position	*b* = −0.63	*SE* = 0.02	Main association not due to overcontrol
ESS without self-rated health	*b* = −0.58	*SE* = 0.02	Main association remains stable
Survey without debt and literacy controls	Indirect effect = −0.15	[−0.18, −0.12]	Mediation not control-dependent
Latent method-factor model	Indirect effect = −0.12	[−0.15, −0.09]	Common-method explanation reduced
Reverse-order SEM	CFI = 0.923	RMSEA = 0.057	Worse fit than hypothesized model

OR, odds ratio; SEM, structural equation model. The ordered-response model was estimated for ESS happiness and life-satisfaction categories. Robustness results are interpreted as sensitivity evidence rather than independent proof of causality.

**Figure 5:**
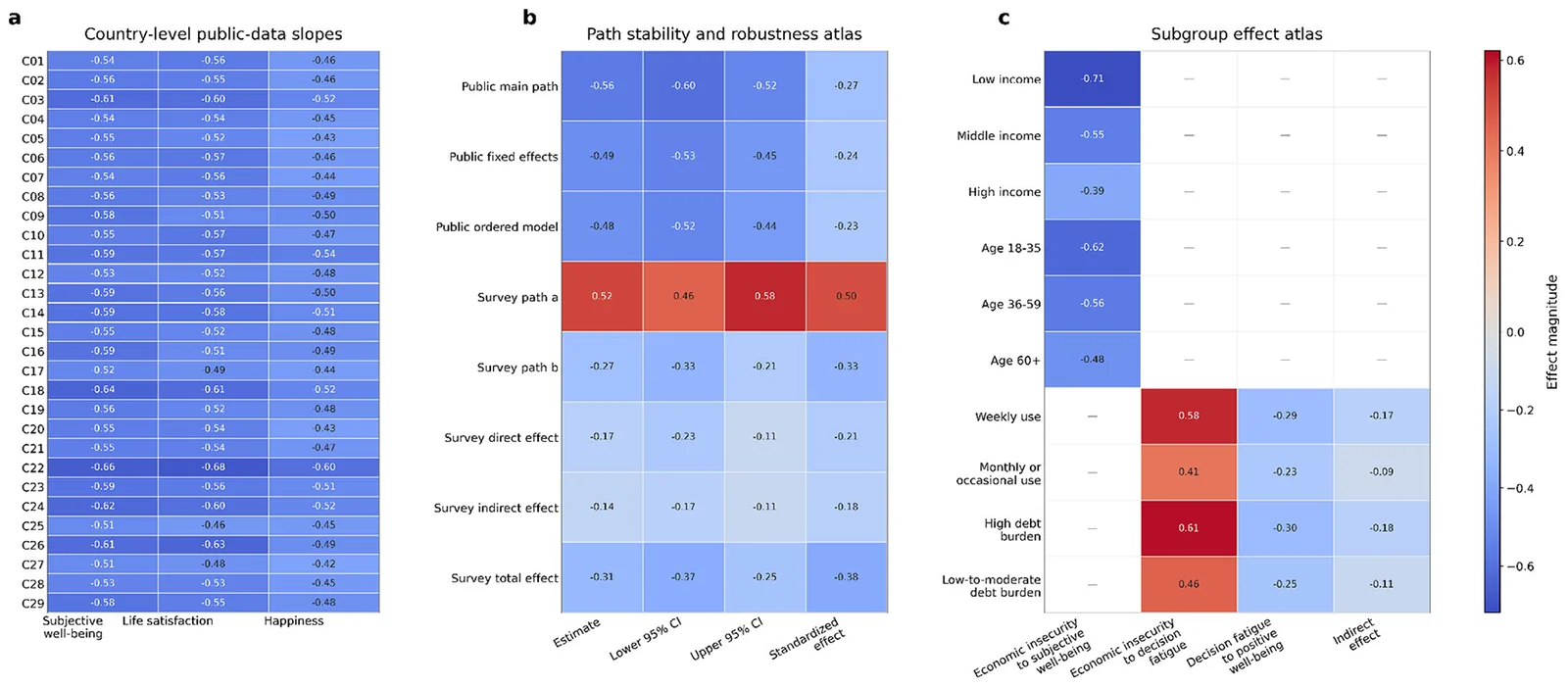
Heatmap atlas of cross-country heterogeneity and effect stability. **(a)** a) Summarizes country-level slopes linking economic insecurity to subjective wellbeing indicators across the 29 ESS countries. **(b)** Compares the magnitude and interval structure of key effects across alternative model specifications. **(c)** integrates subgroup and conditional mediation estimates from the public-data and survey analyses.

Figure 5 provides a final visual synthesis of the empirical results. The country-level panel indicates that the negative association between economic insecurity and subjective wellbeing was not confined to a small set of countries. The specification panel shows that the focal effect was stable when alternative outcomes, controls, and model forms were used. The subgroup panel shows contextual variation without implying that subgroup differences are causal. Together, these analyses support a balanced conclusion: the insecurity–wellbeing association is robust, and the decision-fatigue pathway is stronger in contexts of higher decision demand and heavier financial pressure.

## 5 Discussion

### 5.1 Main findings and psychological interpretation

This study examined whether decision fatigue helps explain why economic insecurity is associated with lower positive wellbeing in contemporary financial life. The ESS analysis showed a robust cross-national association between economic insecurity and lower happiness and life satisfaction. The three-wave survey then showed that economic insecurity was associated with greater financial decision fatigue, and that decision fatigue was associated with lower WHO-5 positive wellbeing. The indirect effect was significant and stable across robustness checks. These findings support the proposed psychological mechanism, while the observational nature of the data requires cautious interpretation.

The results contribute to a more psychologically specific account of economic insecurity. Prior research has shown that financial strain and perceived economic vulnerability are associated with distress and lower wellbeing (Rohde et al., 2016; Kopasker et al., 2018; Ryu and Fan, 2023; Bridges and Disney, 2010). The present study extends this literature by identifying a decision-related process through which insecurity may become burdensome. Economic insecurity appears to be linked not only to worry about resources, but also to a repeated pattern of monitoring, comparison, and effortful financial judgment. This pattern is psychologically important because it can generate fatigue even when individual decisions are routine.

The study also clarifies the role of positive wellbeing. The empirical outcomes used here are happiness, life satisfaction, and WHO-5 wellbeing. These indicators primarily capture subjective and positive affective functioning, not the full eudaimonic structure of psychological wellbeing. This clarification narrows the claim of the study and makes the theoretical contribution more precise: economic insecurity and decision fatigue are associated with lower positive wellbeing, including reduced happiness, life satisfaction, energy, calmness, and vitality.

### 5.2 Digitally mediated financial environments

The results do not imply that digitally mediated financial environments are inherently harmful. Digital financial tools may increase access, convenience, and control for many users. The central point is conditional: when individuals already feel economically insecure, frequent exposure to financial information, options, reminders, and recommendations may increase the number of psychologically salient decision points. This interpretation is supported by the moderation analysis showing that the indirect pathway through decision fatigue was stronger among frequent digital finance users.

This finding helps reconcile two views of digital finance. From an access perspective, digital tools can reduce transaction costs and support financial management. From a psychological-burden perspective, the same tools can increase monitoring pressure and choice exposure. The present study suggests that both may be true. The psychological consequences of digital financial tools depend partly on users' subjective security and financial obligations. For secure users, frequent digital engagement may be manageable. For insecure users, it may become another channel through which financial vulnerability remains cognitively active.

### 5.3 Decision fatigue and intertemporal financial choices

A further implication concerns intertemporal choices. Financial wellbeing depends heavily on decisions that connect the present and the future, including saving, borrowing, debt repayment, insurance, investment, and delayed consumption. Economic insecurity may make these choices more difficult because the individual must weigh immediate relief against future vulnerability. Decision fatigue can intensify this difficulty by reducing the energy available for complex future-oriented evaluation.

The present study did not directly measure delay discounting, saving behavior, debt rollover, or long-term investment choices. Nevertheless, the findings suggest a plausible pathway for future research: when insecurity produces repeated decision fatigue, individuals may become more likely to avoid complex planning, rely on defaults, postpone difficult choices, or prefer short-term relief. Future studies should therefore connect financial decision fatigue with behavioral indicators of intertemporal choice, including savings persistence, credit use, investment horizon, debt repayment regularity, and willingness to delay consumption. Experimental designs could also manipulate financial choice complexity to test whether fatigue changes preferences between immediate and delayed outcomes.

### 5.4 Practical implications

The findings have implications for financial counseling, digital platform design, and wellbeing promotion. Interventions for economically insecure individuals should not only provide information but also reduce unnecessary decision burden. Simplified budgeting routines, pre-commitment tools, default savings settings, and structured decision calendars may help reduce repeated monitoring and comparison. These interventions may be especially useful for people who face heavy debt burdens or who frequently engage with digital finance platforms.

For platform designers, the results suggest caution about equating more information with better support. Personalized prompts, repeated notifications, and continuous performance comparisons may help some users, but they may also increase fatigue among financially insecure users. User-centered design should therefore consider decision load, timing of prompts, clarity of options, and the possibility of low-burden modes for vulnerable users. At the policy level, the findings suggest that economic insecurity should be treated not only as a material issue but also as a psychological burden involving attention, decision demand, and self-regulatory strain.

### 5.5 Limitations and future directions

Several limitations should be acknowledged. First, the ESS component is valuable for external validity but does not measure digital finance use or decision fatigue. It therefore supports only the general association between economic insecurity and positive wellbeing. The digital finance interpretation is tested only in the three-wave survey. Second, although the three-wave design improves temporal ordering, it does not establish causality. All focal constructs are self-reported, and reciprocal relationships remain possible. Third, the WHO-5 at T3 asks about the previous 2 weeks, while the waves were separated by approximately 1 week. This means that the outcome recall window partly overlapped with the preceding waves. The findings should therefore be interpreted as temporally ordered associations rather than conclusive evidence that T2 fatigue caused later wellbeing changes.

Fourth, the financial decision fatigue scale was adapted for this study. The current evidence supports reliability, convergent validity, discriminant validity, and partial measurement invariance, but additional independent validation is needed. Future research should test the scale in other cultural contexts, among older adults, and among populations with different levels of debt and digital finance exposure. Fifth, the heterogeneity analyses used formal interactions and multi-group SEM, but multiple subgroup tests still increase the risk of overinterpretation. The moderation findings should be viewed as theoretically meaningful but in need of replication. Sixth, future research should use longer time intervals, behavioral measures of financial choice, app-use trace data, and experimental or quasi-experimental manipulations of decision complexity to better identify causal pathways.

## 6 Conclusion

The present study examined economic insecurity, decision fatigue, and positive wellbeing in contemporary financial life. The ESS analysis showed that economic insecurity was consistently associated with lower happiness and life satisfaction across 29 countries. The three-wave survey of digital finance users further showed that economic insecurity was associated with greater financial decision fatigue, which in turn was associated with lower WHO-5 positive wellbeing. The mediation pathway remained stable across multiple robustness checks and was stronger among frequent digital finance users and participants with heavier debt burdens.

These findings suggest that economic insecurity is psychologically consequential not only because it reflects limited resources, but also because it is associated with a more fatiguing pattern of financial decision engagement. In digitally mediated financial environments, financially vulnerable individuals may face repeated prompts to monitor, compare, and decide. Such repeated decision demands may reduce energy, calmness, and positive functioning. A psychologically informed approach to economic insecurity should therefore consider both material vulnerability and the decision burden imposed by everyday financial life.

## Data Availability

The raw data supporting the conclusions of this article will be made available by the authors, without undue reservation.

## References

[B1] Adler N. E. Epel E. S. Castellazzo G. Ickovics J. R. (2000). Relationship of subjective and objective social status with psychological and physiological functioning: preliminary data in healthy white women. Health Psychol. 19, 586–592. doi: 10.1037/0278-6133.19.6.586 11129362

[B2] Arnold M. Goldschmitt M. Rigotti T. (2023). Dealing with information overload: a comprehensive review. Front. Psychol. 14:1122200. doi: 10.3389/fpsyg.2023.1122200 37416535 PMC10322198

[B3] Basyouni S. S. El Keshky M. E. S. (2021). Job insecurity, work-related flow, and financial anxiety in the midst of COVID-19 pandemic and economic downturn. Front. Psychol. 12:632265. doi: 10.3389/fpsyg.2021.632265 34335356 PMC8320320

[B4] Belanche D. Casaló L. V Flavián C. (2019). Artificial intelligence in FinTech: understanding robo-advisors adoption among customers. Indus. Manag. Data Syst. 119, 1411–1430. doi: 10.1108/IMDS-08-2018-0368

[B5] Boksem M. A. S. Tops M. (2008). Mental fatigue: costs and benefits. Brain Res. Rev. 59, 125–139. doi: 10.1016/j.brainresrev.2008.07.001 18652844

[B6] Bridges S. Disney R. (2010). Debt and depression. J. Health Econ. 29, 388–403. doi: 10.1016/j.jhealeco.2010.02.003 20338649

[B7] Carlin B. Olafsson A. Pagel M. (2023). Mobile apps and financial decision making. Rev. Finan. 27, 977–996. doi: 10.1093/rof/rfac040

[B8] De Witte H. Pienaar J. De Cuyper N. (2016). Review of 30 years of longitudinal studies on the association between job insecurity and health and well-being: is there causal evidence? Aust. Psychol. 51, 18–31. doi: 10.1111/ap.12176

[B9] Diener E. Suh E. M. Lucas R. E. Smith H. L. (1999). Subjective well-being: three decades of progress. Psychol. Bull. 125, 276–302. doi: 10.1037/0033-2909.125.2.276

[B10] Eppler M. J. Mengis J. (2004). The concept of information overload: a review of literature from organization science, accounting, marketing, MIS, and related disciplines. Inform. Soc. 20, 325–344. doi: 10.1080/01972240490507974

[B11] Hagger M. S. Wood C. Stiff C. Chatzisarantis N. L. D. (2010). Ego depletion and the strength model of self-control: a meta-analysis. Psychol. Bull. 136, 495–525. doi: 10.1037/a0019486 20565167

[B12] Haushofer J. Fehr E. (2014). On the psychology of poverty. Science 344, 862–867. doi: 10.1126/science.1232491 24855262

[B13] Huppert F. A. So T. T. C. (2013). Flourishing across Europe: application of a new conceptual framework for defining well-being. Soc. Indic. Res. 110, 837–861. doi: 10.1007/s11205-011-9966-7 23329863 PMC3545194

[B14] Inzlicht M. Schmeichel B. J. Macrae C. N. (2014). Why self-control seems (but may not be) limited. Trends Cogn. Sci. 18, 127–133. doi: 10.1016/j.tics.2013.12.009 24439530

[B15] Keyes C. L. M. (2002). The mental health continuum: from languishing to flourishing in life. J. Health Soc. Behav. 43, 207–222. doi: 10.2307/3090197 12096700

[B16] Kopasker D. Montagna C. Bender K. A. (2018). Economic insecurity: a socioeconomic determinant of mental health. SSM – Popul. Health 6, 184–194. doi: 10.1016/j.ssmph.2018.09.006 30417065 PMC6215053

[B17] Lei X. Shen Y. Yang L. (2023). Digital financial inclusion and subjective well-being-Evidence from China health and retirement longitudinal study. China Econ. Rev. 81:102013. doi: 10.1016/j.chieco.2023.102013

[B18] Mani A. Mullainathan S. Shafir E. Zhao J. (2013). Poverty impedes cognitive function. Science 341, 976–980. doi: 10.1126/science.1238041 23990553

[B19] Marsh E. Perez Vallejos E. Spence A. (2024). Overloaded by information or worried about missing out on it: a quantitative study of stress, burnout, and mental health implications in the digital workplace. SAGE Open 14, 1–17. doi: 10.1177/21582440241268830

[B20] Müller T. Apps M. A. J. (2019). Motivational fatigue: a neurocognitive framework for the impact of effortful exertion on subsequent motivation. Neuropsychologia 123, 141–151. doi: 10.1016/j.neuropsychologia.2018.04.030 29738794

[B21] Pignatiello G. A. Martin R. J. Hickman R. L. (2020). Decision fatigue: a conceptual analysis. J. Health Psychol. 25, 123–135. doi: 10.1177/1359105318763510 29569950 PMC6119549

[B22] Polman E. Vohs K. D. (2016). Decision fatigue, choosing for others, and self-construal. Soc. Psychol. Personal. Sci. 7, 471–478. doi: 10.1177/1948550616639648

[B23] Rohde N. Tang K. K. Osberg L. Rao P. (2016). The effect of economic insecurity on mental health: recent evidence from Australian panel data. Soc. Sci. Med. 151, 250–258. doi: 10.1016/j.socscimed.2015.12.014 26826683

[B24] Ryff C. D. (1989). Happiness is everything, or is it? Explorations on the meaning of psychological well-being. J. Pers. Soc. Psychol. 57, 1069–1081. doi: 10.1037/0022-3514.57.6.1069

[B25] Ryff C. D. Keyes C. L. M. (1995). The structure of psychological well-being revisited. J. Pers. Soc. Psychol. 69, 719–727. doi: 10.1037/0022-3514.69.4.719 7473027

[B26] Ryu S. Fan L. (2023). The relationship between financial worries and psychological distress among U.S. adults. J. Fam. Econ. Issues 44, 16–33. doi: 10.1007/s10834-022-09820-9 35125855 PMC8806009

[B27] Shahrzadi L. Mansouri A. Alavi M. Shabani A. (2024). Causes, consequences, and strategies to deal with information overload: a scoping review. Int. J. Inform. Manag. Data Insights 4:100261. doi: 10.1016/j.jjimei.2024.100261

[B28] Singh-Manoux A. Adler N. E. Marmot M. G. (2003). Subjective social status: its determinants and its association with measures of ill-health in the Whitehall II study. Soc. Sci. Med. 56, 1321–1333. doi: 10.1016/S0277-9536(02)00131-4 12600368

[B29] Smith H. J. Pettigrew T. F. Pippin G. M. Bialosiewicz S. (2012). Relative deprivation: a theoretical and meta-analytic review. Pers. Soc. Psychol. Rev. 16, 203–232. doi: 10.1177/1088868311430825 22194251

[B30] Topp C. W. Østergaard S. D. Søndergaard S. Bech P. (2015). The WHO-5 Well-Being Index: a systematic review of the literature. Psychother. Psychosom. 84, 167–176. doi: 10.1159/000376585 25831962

[B31] Vohs K. D. Baumeister R. F. Schmeichel B. J. Twenge J. M. Nelson N. M. Tice D. M. (2008). Making choices impairs subsequent self-control: a limited-resource account of decision making, self-regulation, and active initiative. J. Pers. Soc. Psychol. 94, 883–898. doi: 10.1037/0022-3514.94.5.883 18444745

[B32] Zhao C. Li X. Yan J. (2024). The effect of digital finance on residents' happiness: the case of mobile payments in China. Electr. Comm. Res. 24, 69–104. doi: 10.1007/s10660-022-09549-5

